# Controlled composition of Zn-doped iron oxide nanoparticles for optimised MPI performance

**DOI:** 10.1039/d6na00526h

**Published:** 2026-09-09

**Authors:** Deandra L. Pragasam, Emma E. Brotherton, Agus R. Poerwoprajitno, Zeno R. Ramadhan, Brendan Lee, Andre Bongers, Kartika Wardhani, Dale L. Huber, Richard D. Tilley

**Affiliations:** a School of Chemistry, University of New South Wales Sydney NSW 2052 Australia r.tilley@unsw.edu.au; b Center for Integrated Nanotechnologies, Sandia National Laboratories Albuquerque NM 87185 USA arpoerw@sandia.gov; c Electron Microscope Unit, Mark Wainwright Analytical Centre, University of New South Wales Sydney NSW 2052 Australia; d Biological Resources Imaging Laboratory, Mark Wainwright Analytical Centre, University of New South Wales Sydney NSW 2052 Australia

## Abstract

Nanoparticle composition and crystal phase critically affect magnetic particle imaging performance by influencing magnetic properties. Compositional optimisation can be based on the iron oxide phase and potential dopants. Here, we show that 13 nm zinc-doped maghemite nanoparticles exhibit MPI signal intensity comparable to that of a 25 nm commercial tracer. Their smaller size and maghemite crystal phase make them promising alternative tracers for future MPI applications.

## Abstract 2

Nanoparticle composition and crystal phase critically affect magnetic particle imaging performance by influencing magnetic properties.

## Graphical abstract

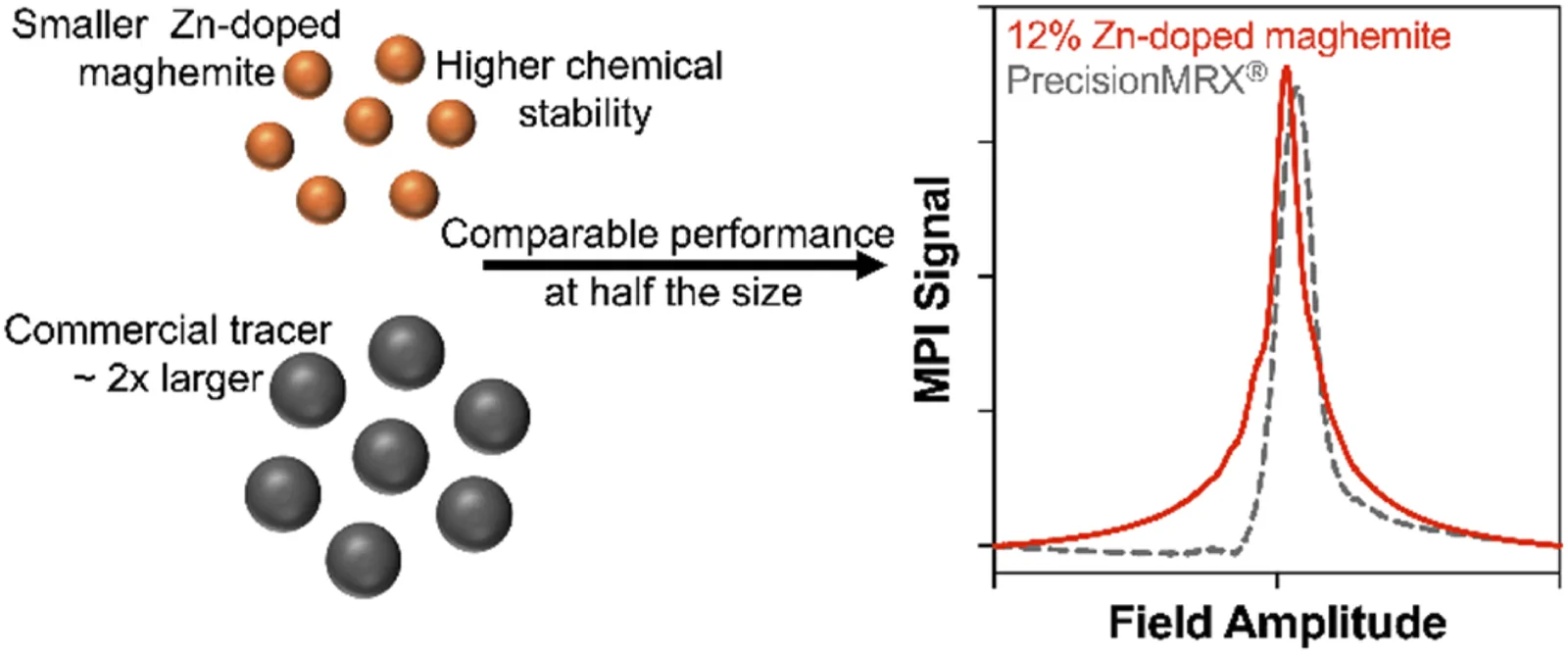

Magnetic particle imaging (MPI) has emerged as a highly quantitative tomographic modality capable of real-time, *in vivo* tracking of superparamagnetic nanoparticles with no background signal from soft tissue.^1–3^ Unlike magnetic resonance imaging, which detects proton relaxation, MPI directly measures the nonlinear magnetisation response of a magnetic tracer to a spatially varying, oscillating field. Because the image is generated solely by the tracer, MPI offers a linear concentration response, high sensitivity, and depth-independent contrast, making it attractive for vascular imaging, cell tracking, and image-guided magnetic therapies.^4–6^ It also means that MPI performance is limited by the tracer itself, and optimised magnetic nanoparticles are now widely recognized as the principal bottleneck to clinical translation.^7–9^

The MPI signal is derived from the field derivative of the magnetisation. Therefore, both the signal amplitude and spatial resolution depend on how sharply and quickly the particle magnetic moment reverses as the field-free region passes. Both the saturation magnetisation (*M*_sat_), which sets the maximum achievable signal, and the relaxation dynamics, governed by the effective magnetic anisotropy, core size and size distribution, and colloidal state, determine the MPI tracer performance.^7,9,10^

Magnetite (Fe_3_O_4_) is the most widely used tracer material, but it contains Fe^2+^ and is metastable under ambient and physiological conditions. Progressive oxidation during storage alters core size, anisotropy and relaxation dynamics, causing MPI signal to change over time.^7,9,10^ Maghemite (Fe_2_O_3_), the fully oxidised end member, contains only Fe^3+^ and cannot oxidise further, providing a stable basis for reproducible tracing, at the cost of a lower *M*_sat_, since cation vacancies on the octahedral sublattice reduce the net moment. Since MPI tracer performance depends on the magnetic properties of the nanoparticles, it is important to evaluate how Zn doping influences them.

In this work, we present a synthetic strategy that enables precise control over Zn^2+^ incorporation and deliberate tuning of the magnetite–maghemite phase balance in Zn-doped iron oxide nanoparticles. We systematically investigate how compositional modulation influences MPI tracer performance by examining the effects of Zn doping levels, particle oxidation states, and phase fractions on *M*_sat_, magnetic relaxation dynamics, and ultimately MPI signal amplitude and spatial resolution. Our findings link nanocrystal composition and structure to MPI sensitivity, showing that 12 at% Zn-doped maghemite generates a comparable MPI signal than the commercial PrecisionMRX® standard at approximately half the particle diameter, with a point spread function FWHM of 22.4 mT. This advantage does not arise from increased *M*_sat_, but from low blocking temperature, which keeps the particles fully superparamagnetic and therefore dynamically responsive at the 45 kHz drive frequency.^11–14^

A series of Zn-doped maghemite nanoparticles containing between 3 and 12 at% Zn were synthesised *via* a two-step route: thermal decomposition to form magnetite, followed by controlled oxidation to maghemite. Firstly, magnetite nanoparticles were synthesised *via* thermal decomposition of iron(iii) acetylacetonate and zinc(ii) acetylacetonate in benzyl ether at 300 °C. Oleic acid and oleylamine were utilised as co-surfactants to control nanoparticle growth. Next, these magnetite nanoparticles were oxidised to maghemite nanoparticles using trimethylamine *N*-oxide (TMAO) as a mild oxidising agent. This synthetic approach differs from conventional air oxidation and direct maghemite synthesis, which often produce polycrystalline nanoparticles with varying ratios of maghemite and magnetite.^9,15,16^ In contrast, oxidising with TMAO maintains high crystallinity and phase purity of the nanoparticles.^16^

The morphology and size distribution of the nanoparticles were determined using transmission electron microscopy (TEM, Fig. 1a, c, e and g). All nanoparticles had a spherical morphology, as seen in the TEM images and their corresponding histograms (Fig. 1b, d, f and h), an average diameter of 13.0 nm was achieved for all nanoparticle compositions. Furthermore, the uniformity of the nanoparticles was maintained across the series, with a standard deviation of ∼1.7 nm for all nanoparticles. Upon oxidation, the morphology of the nanoparticles remained roughly spherical, and there was no observable change in nanoparticle diameter (Fig. 1). Similarly, all nanoparticles had a diameter of ∼12.6 nm with a standard deviation of ∼1.5 nm. These results confirm that the two-step approach maintains the morphology and narrow size dispersity of the nanoparticles. The final nanoparticle diameter for all nanoparticle compositions falls below the critical diameter (25 nm) for superparamagnetic behaviour near ambient temperatures, which is critical for MPI tracer performance.^4,5^ The energy-dispersive X-ray spectroscopy (EDS) elemental mapping of Fe and Zn demonstrates a homogeneous distribution of Zn throughout the magnetite and maghemite nanoparticles (Fig. 1i). Importantly, the oxidised maghemite nanoparticles also retained a uniform Zn distribution post-oxidation (Fig. S1). This indicates that Zn remains within the crystal structure post-oxidation (Fig. S1).

**Fig. 1:**
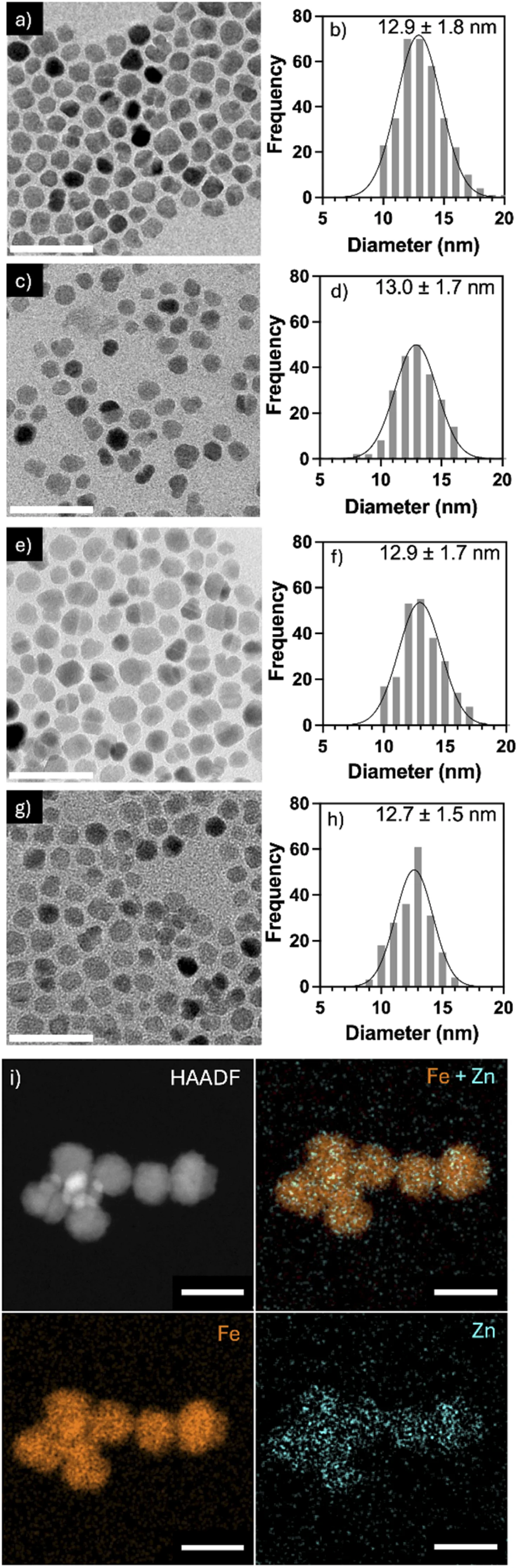
TEM images with corresponding size-distribution histograms for: (a and b) 0 at% Zn magnetite, (c and d) 12 at% Zn magnetite, (e and f) 0 at% Zn maghemite, and (g and h) 12 at% Zn maghemite. The scale bar in each image represents 50 nm. (i) EDS elemental maps of 12 at% Zn magnetite as a representation of dopant distribution. All EDS maps have a scale bar of 20 nm.

The crystal structure and crystallite size of the nanoparticles were determined using X-ray diffraction (XRD). The XRD patterns of the nanoparticles are in good agreement with the reference crystal structures of magnetite, Zn-doped magnetite and maghemite (Fig. S2, SI).^17–19^ The lattice constants of both undoped and doped nanoparticles were determined from the (311) XRD peak. The calculated lattice constant of magnetite increases from 8.382 (0 at% Zn) to 8.414 Å (12 at% Zn) (Table S1). This confirms the incorporation of Zn into the crystal structure as the larger Zn^2+^ cations (0.74 Å) replace the smaller Fe^3+^ ions (0.64 Å) in the T_d_ sites, causing a slight expansion of the lattice parameters. Similarly, the lattice constants for 0 and 12 at% Zn maghemite nanoparticles also increased from 8.382 to 8.418 Å.^5,17,19^

The final nanoparticle compositions were determined by inductively coupled plasma optical emission spectroscopy (ICP-OES). Magnetite nanoparticles were found to contain 0, 3, 7, and 12 at% Zn. An aliquot of magnetite nanoparticles was oxidised to maghemite nanoparticles using TMAO under mild conditions to form a series of Zn-doped maghemite nanoparticles with Zn contents of 0, 3, 7, and 12 at% as determined by ICP-OES.^21,22^

The MPI tracer performance of the Zn-doped nanoparticles was evaluated by comparing the point spread function (PSF) of the MPI signal for both undoped and Zn-doped nanoparticles with a commercial reference tracer, PrecisionMRX® (Fig. 2, S3 and Table S2). PrecisionMRX® consists of spherical, undoped single-core magnetite nanoparticles with a reported size of 25 ± 2 nm, engineered for MPI applications. The PrecisionMRX® tracer exhibits a normalised signal intensity and full width at half maximum (FWHM) of 0.96 a.u. and 15.1 mT, respectively. As shown in Fig. 2, magnetite nanoparticles containing 0 and 12 at% Zn, and maghemite nanoparticles containing 0 and 12 at% Zn generated normalised signal intensities of 0.76, 0.95, 0.61, and 1.0 a.u., respectively, with corresponding FWHM values of 26.2, 21.7, 18.4, and 22.4 mT, respectively (Table S2). The observed enhancement in signal intensity is consistent with the previously explained mechanism of Zn^2+^ incorporation.^5,8,19^ The strongest MPI signal was obtained for 12 at% Zn maghemite, which showed a slight increase in signal intensity, with a narrow FWHM of 22.4 mT which is comparable to that of the commercial standard. This demonstrates that the combination of Zn incorporation and controlled oxidation yields nanoparticles with optimised MPI signal intensity. Notably, 12 at% Zn maghemite matches the MPI performance of the commercial tracer at approximately half the particle diameter.

**Fig. 2:**
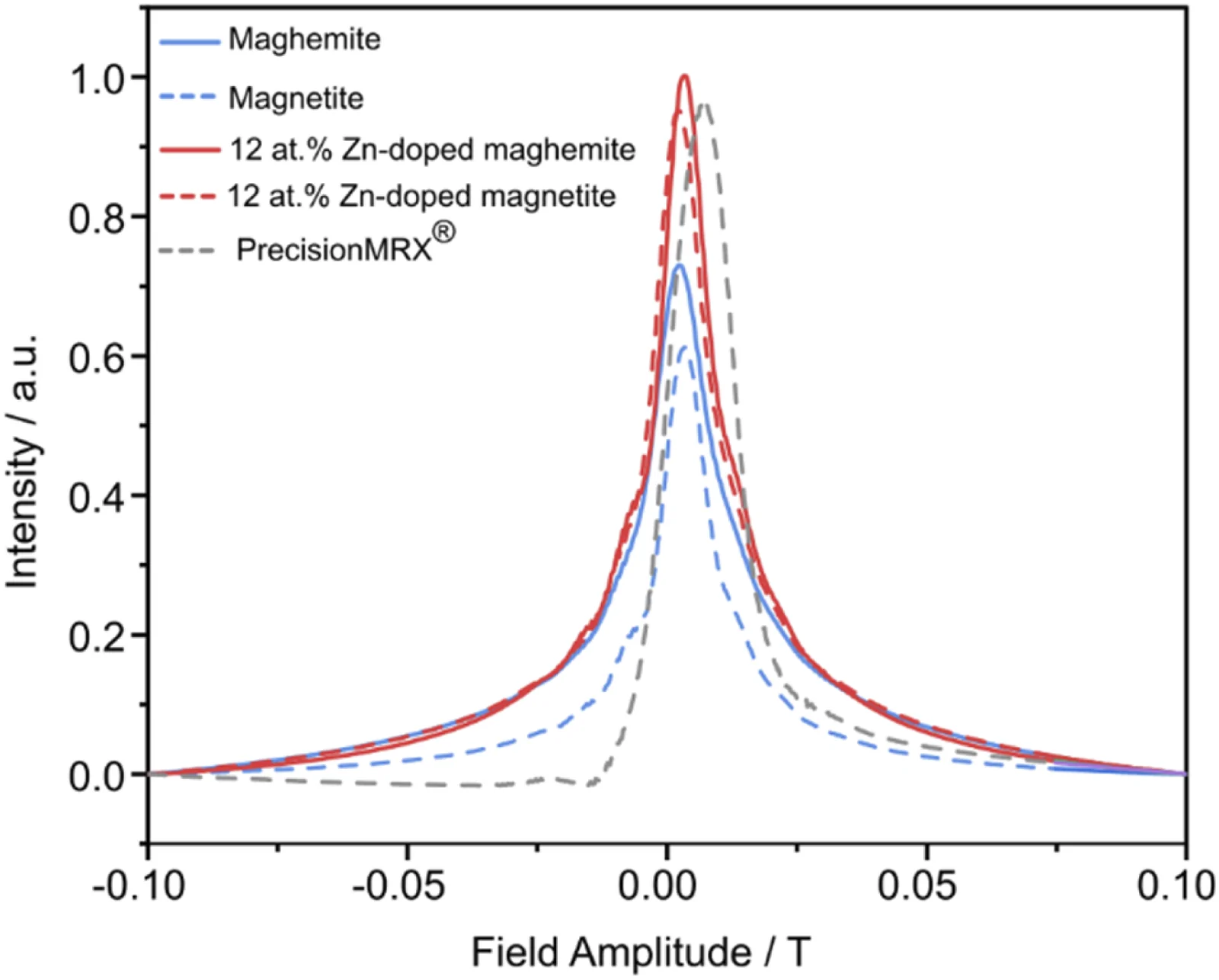
Normalised point spread function for the magnetite (dashed blue), maghemite (solid blue), 12 at% Zn-doped magnetite (dashed red), 12 at% Zn-doped maghemite (solid red) and PrecisionMRX® (dashed grey). The value was normalized by the mass of the nanoparticle.

To better understand the high MPI signal intensity of the Zn-doped nanoparticles, magnetic properties of the nanoparticles were characterised at 300 K using a vibrating sample magnetometer (VSM) to obtain hysteresis loops (Fig. 3a and S3). The magnetic moment was normalised by the mass of the nanoparticles. All samples exhibited superparamagnetic behaviour, with coercivity values below 5 Oe and magnetic remanence below 3 emu g_NP_^−1^ (Table S2). These magnetic properties are consistent with iron oxide nanoparticles designed for MPI signal generation.^1,2,7,10,20,23,24^

The *M*_sat_ of the 12 at% Zn-doped magnetite and maghemite nanoparticles are lower than that of undoped magnetite and the commercial PrecisionMRX® tracer. Therefore, the improved PSF intensity cannot be attributed to a higher *M*_sat_. We next performed alternating-current (AC) susceptibility measurements over frequencies from 30 Hz to 10 kHz (Fig. 3b and c) and analysed the frequency-dependent blocking temperature using the Néel–Brown relaxation model. The blocking temperature at our highest obtainable frequency of 10 kHz were 218 K for our 12 at% Zn-doped maghemite and 281 K for the commercial reference. To get an indication for the performance at the actual MPI operating frequency of 45 kHz we estimated the values to this range, yielding blocking temperature of 228 K and 302 K, for the doped particle and PrecisionMRX®, respectively. The details of our blocking temperature calculation are presented in Fig. S4 (SI).^23^ Thus, in our room temperature MPI measurements, the 12 at% Zn-doped maghemite remains well within the superparamagnetic regime, while the PrecisionMRX® reference appears to be in a partially blocked regime. The high blocking temperature of the PrecisionMRX® attributed to its larger average size of 25 nm.

**Fig. 3:**
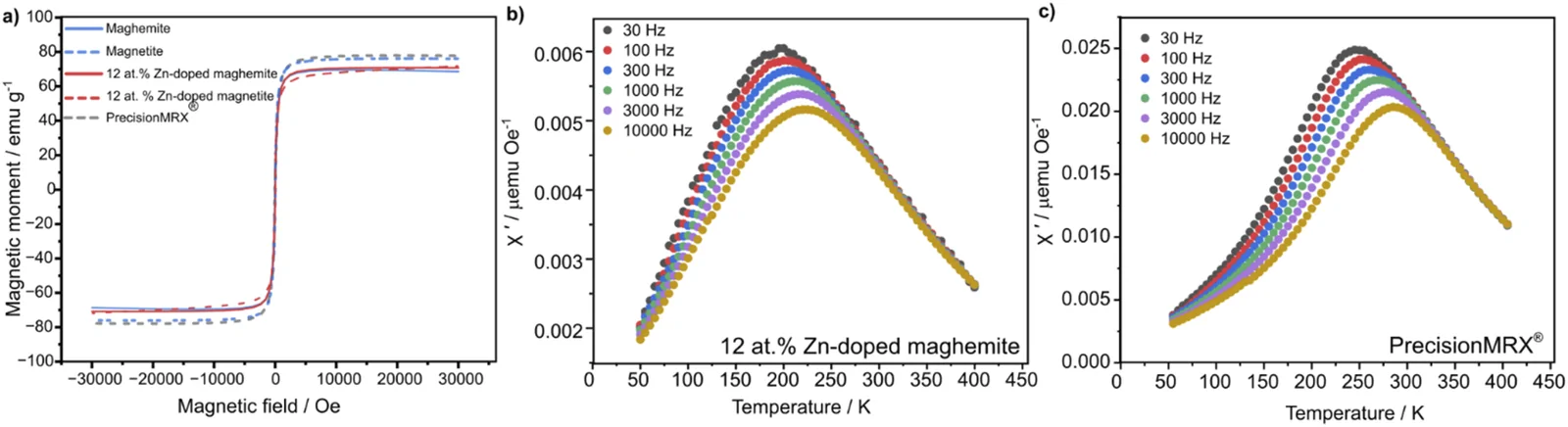
(a) The hysteresis loop of magnetic nanoparticles at 300 K using vibrating sample magnetometer. The value was normalised by the mass of the nanoparticle. (b and c) Alternating current (AC) susceptibility measurement of (b) 12 at% Zn-doped maghemite and (c) PrecisionMRX® nanoparticle as a function of temperature at different frequencies, reported as the real, in-phase component of the complex susceptibility, *χ*′.

The presence of Zn doping can modify the cation distribution in the spinel lattice, affect Fe–O–Fe superexchange interactions, and change the degree of spin disorder and surface anisotropy, thereby altering the magnetocrystalline anisotropy. The magnetocrystalline anisotropy of 12 at% Zn-doped maghemite is 9.86 × 10^4^ J m^−3^, lower than that of undoped maghemite, 1.06 × 10^5^ J m^−3^. This reduced anisotropy indicates a lower energy barrier for rotation or reversal of the nanoparticle magnetic moment, enabling a more facile response to an alternating magnetic field. This is consistent with the observed decrease in blocking temperature (Fig. S5, SI), where 12 at% Zn-doped maghemite exhibits a blocking temperature 23 K lower than that of undoped maghemite.

The smaller size and maghemite crystal phase of the 12 at% Zn-doped maghemite provide an attractive alternative to the PrecisionMRX® commercial MPI tracer. At the same time, the maghemite phase is structurally stable due to its oxidised iron state, which can reduce susceptibility to phase transformation and chemical degradation during storage.

## Conclusions

Overall, Zn incorporation combined with controlled oxidation to the maghemite phase produced a tracer that delivers MPI signal intensity exceeding the commercial PrecisionMRX® standard. Among all samples, the 12 at% Zn-doped maghemite generated the highest normalised MPI signal, despite having an approximately two-fold smaller particle diameter than PrecisionMRX®. However, the spatial resolution of the 12 at% Zn-doped maghemite is slightly lower than PrecisionMRX®. Magnetic characterisation indicates this performance is not driven by higher *M*_sat_, but instead by more favourable relaxation behaviour. AC susceptibility analysis revealed that 12 at% Zn-doped maghemite has a substantially lower blocking temperature, indicating it remains fully superparamagnetic under MPI operating conditions, whereas PrecisionMRX® is expected to be partially blocked at 300 K. This suggests that the larger particles cannot fully follow the 45 kHz excitation field in MPI due to slow Néel relaxation, while Zn doping maintains fast magnetic relaxation in the smaller particles. As a result, the Zn-doped particles remain dynamically responsive, enabling them to generate a comparable or even stronger MPI signal despite their lower magnetic moment arising from their smaller core size. This frequency-dependent relaxation advantage suggests that 12 at% Zn-doped maghemite is a promising tracer candidate for future MPI applications.

## Author contributions

The manuscript was written through contributions of all authors. All authors have given approval to the final version of the manuscript.

## Conflicts of interest

There are no conflicts to declare.

## Supplementary Material

NA-008-D6NA00526H-s001

## Data Availability

The data supporting this article have been included as part of the supplementary information (SI). Supplementary information: includes TEM images, XRD pattern, summary of magnetic and MPI properties, and extrapolation of blocking temperature. See DOI: https://doi.org/10.1039/d6na00526h.
