# Supplementary material for: Controlled composition of Zn-doped iron oxide nanoparticles for optimised MPI performance
Source: Nanoscale Adv. 2026 Sep 9;8(22):6582–6. doi: 10.1039/d6na00526h (PMC13585089; doi:10.1039/d6na00526h)
Supplement: Supplementary file 1 [file NA-008-D6NA00526H-s001.pdf]

Supplementary Information (SI) for Nanoscale Advances.
This journal is © The Royal Society of Chemistry 2026

Supplementary Information

Controlled Composition of Zn-Doped Iron Oxide Nanoparticles for Optimised MPI Performance

Deandra L. Pragasam[a], Emma E. Brotherton[a], Agus R. Poerwoprajitno*[b], Zeno R. Ramadhan[c], Brendan Lee[d], Andre Bongers[d], Kartika Wardhani[b], Dale L. Huber[b], Richard D. Tilley*[a,c]

a. *School of Chemistry, The University of New South Wales, Sydney, New South Wales, 2052, Australia.*
b. *Center for Integrated Nanotechnologies, Sandia National Laboratories, Albuquerque, New Mexico 87123, United States*
c. *Electron Microscope Unit, Mark Wainwright Analytical Centre, The University of New South Wales, Sydney, NSW 2052, Australia*
d. *Biological Resources Imaging Laboratory, Mark Wainwright Analytical Centre, UNSW Sydney, Sydney, NSW 2052, Australia*

*email: r.tilley@unsw.edu.au (RDT), arpoerw@sandia.gov (ARP)

Experimental:

Chemicals: All reagents were used as received unless stated otherwise. Iron (III) acetylacetonate ($Fe(acac)_3$; 97%), zinc (II) acetylacetonate ($Zn(acac)_2$; 99.9%), oleylamine (70%), oleic acid (90%), benzyl ether (98%) and trimethylamine N-oxide (TMAO; 98%) were purchased from Sigma-Aldrich Pty Ltd, Australia. Ethanol (96%), toluene (99.9%), nitric acid (37%), and hydrochloric acid (70%) were purchased from Chem-Supply Pty. Ltd, Australia.

Synthesis of Zn-doped magnetite nanoparticles

A typical synthesis protocol is as follows, $Fe(acac)_3$ (639.1 mg, 1.81 mmol) and $Zn(acac)_2$ (71.3 mg, 0.27 mmol) were added to a multi-neck round bottom flask. Oleylamine (1.2 mL, 3.65 mmol), oleic acid (1.22 mL, 4.23 mmol) and benzyl ether (10 mL) were added. The reaction solution was magnetically stirred and degassed using a vacuum for 90 minutes at 120 °C. Under a continuous flow of argon, the solution was heated to 200 °C at 2 °C/min and held at this temperature for 120 minutes. The reaction was then heated to 300 °C at 2 °C/min and refluxed for 60 minutes. After refluxing, the reaction mixture was allowed to cool to room temperature. The reaction mixture was transferred to a falcon tube containing ethanol (30 mL) and toluene (2 mL). The mixture was centrifuged for 5 minutes at 1600 relative centrifugal force (rcf) and the supernatant was discarded. The nanoparticles were redispersed in toluene (8 mL) and oleylamine (30 mL) via sonication and centrifuged for 3 minutes at 650 rcf, discarding the supernatant. The nanoparticles were redispersed in ethanol (4 mL) via sonication and centrifuged for 5 minutes at 1600 rcf. Magnetite nanoparticles were collected and suspended in toluene (2 mL) for storage at room temperature.

Oxidation of Zn-doped magnetite nanoparticles

For the oxidation of maghemite, trimethylamine N-oxide (285.4 mg, 3.8 mmol) was dissolved in a mixture of benzyl ether (5 mL), oleic acid (0.75 mL, 2.36 mmol), and a 1 M solution of magnetite in benzyl ether (0.5 mL) in a multi-neck round bottom flask. The mixture was degassed for 60 minutes at 100 °C. The temperature was increased to 135 °C, for 6 hours. The reaction was allowed to cool to room temperature. The brown-orange dispersion was transferred to a falcon centrifuge tube containing a 1:1 ethanol/toluene mixture (20 mL). The solution was magnetically cleaned repeatedly until the supernatant was clear. The maghemite nanoparticles were redispersed in toluene (2 mL) for storage at room temperature.

Characterisation Methods:

Transmission Electron Microscopy (TEM)

TEM images were captured using a JEOL F200 with a 200 KeV emission. To prepare the TEM grids 10 µL of a dilute nanoparticle dispersion was drop-cast onto a carbon-coated copper grid obtained from Ted Pella, Inc. and allowed to evaporate at room temperature. *Image J* software was used to determine the size distribution of nanoparticles.

For EDS elemental mapping, a dilute nanoparticle dispersion was first drop-cast onto a TEM grid, followed by dropping hot ethanol (60 °C) to remove excess surfactant. After 20 s, excess ethanol was wicked away using filter paper. The grid was allowed to dry at room temperature.

X-ray diffraction (XRD)

XRD was conducted on a PANalytical Empyrean II XRD system with a cobalt radiation source (λ = 1.790 Å). The system operated at 40 mA and 45 kV, using a 1⁄4 divergence slit, 10 mm bronze mask and 1⁄2 anti-scatter slit to define the beam. The diffraction pattern was collected from 10° to 120° 2θ with a step size of 0.026°, and time per step of 425 seconds at room temperature. Particles were drop-casted into the 10 mm cavity of the 30 mm zero-background diffraction plate until an opaque layer was formed.

Crystallite size was calculated using the Scherrer equation as described below:

$$D = \frac{K\,\lambda}{\beta \cos\theta}$$

Where:

D is the crystallite size (nm), K is the shape factor (0.94), λ is is the X-ray wavelength (Co Kα = 1.79 Å), β is the full width half maximum (FWHM) of the diffraction peak, and θ is the Bragg angle.

Lattice constant was calculated as describe below for a face cubic centred (fcc) structure:

$$a = d_{hkl}\sqrt{h^2 + k^2 + l^2}$$

$$d_{hkl} = \frac{\lambda}{2 \sin\theta}$$

Where**:**

a is the lattice constant. d_{hkl} is the lattice spacing of specific plane, h, k, and l are the miller indices of the peak, λ is the X-ray wavelength (Co Kα = 1.79 Å), and θ is Bragg angle.

Inductively Coupled Plasma Optical Emission Spectroscopy (ICP-OES)

ICP-OES was used to determine the concentration of iron and zinc using an Optima7000 PerkinElmer. A 200 µL aliquot of nanoparticles were dried with nitrogen gas and digested in aqua regia (1 mL) containing 3 parts hydrochloric acid and 1 part nitric acid. The solution was heated to 80 °C for 1 hour. After an hour, Milli-Q water (9 mL) was added to dilute the sample before analysis.

Vibrating Sample Magnetometer (VSM)

Magnetic characterisation was conducted using a vibrating sample magnetometer (VSM) to investigate the field-dependent magnetic properties of the sample. Prior to measurement, the diluted nanoparticle solution was dropped directly onto a cotton swab without further purification, allowing the nanoparticles to rapidly spread across the substrate and thereby minimising particle–particle interactions. The cotton swab was placed on the quartz paddle sample holder (Quantum Design 4096-392). Hysteresis loops were obtained at 300 K as the magnetic field cycled between -30 and +30 kOe, with the magnetic field stabilised at each data point. The magnetic measurements were normalised to the total mass of nanoparticles. The diamagnetic contribution was subtracted from the hysteresis curve shown in Figure 3a. The AC measurements were conducted from 50 to 400 K at frequencies of 30, 100, 300, 1000, 3000, and 10000 Hz. The blocking temperature was determined by choosing the highest AC susceptibility in each frequency.

Magnetic Particle Imaging Measurements

The MPI performance of nanoparticles was evaluated using 2D projection scan utilising a preclinical MPI scanner (Magnetic Insight Inc., Momentum). Each sample was scanned using a relaxometry module equipped to the scanner and subjected to a constant frequency of 45 kHz and a focusing gradient strength of 5.7 T/m to produce the MPI signal at room temperature. Briefly, the response of the NPs to the 45 kHz drive field was recorded in a 1 mL volume of n-hexane as the solvent. The MPI intensity was normalised by the mass of the nanoparticle.

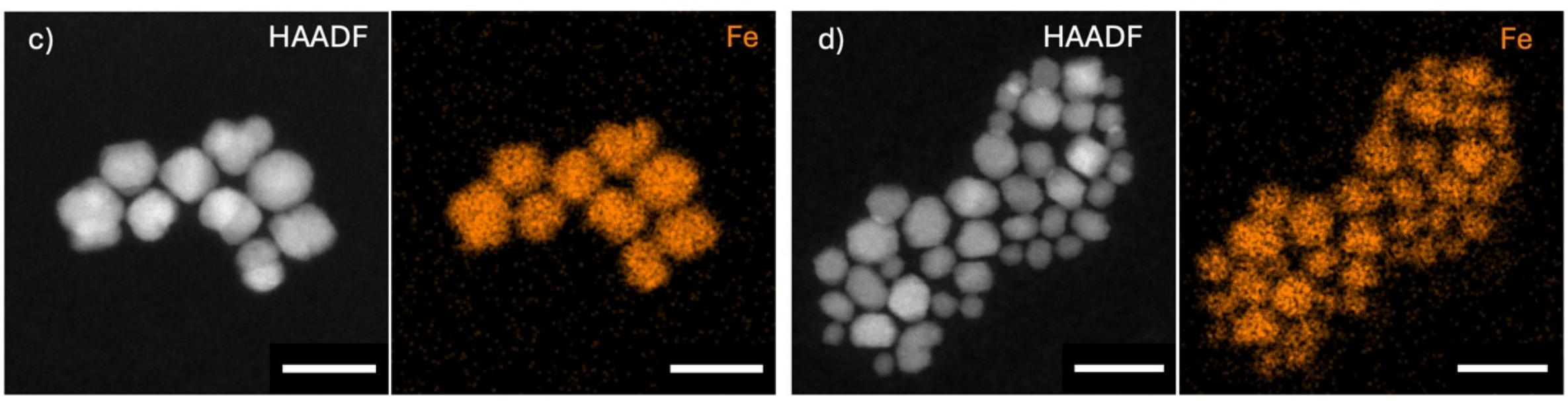

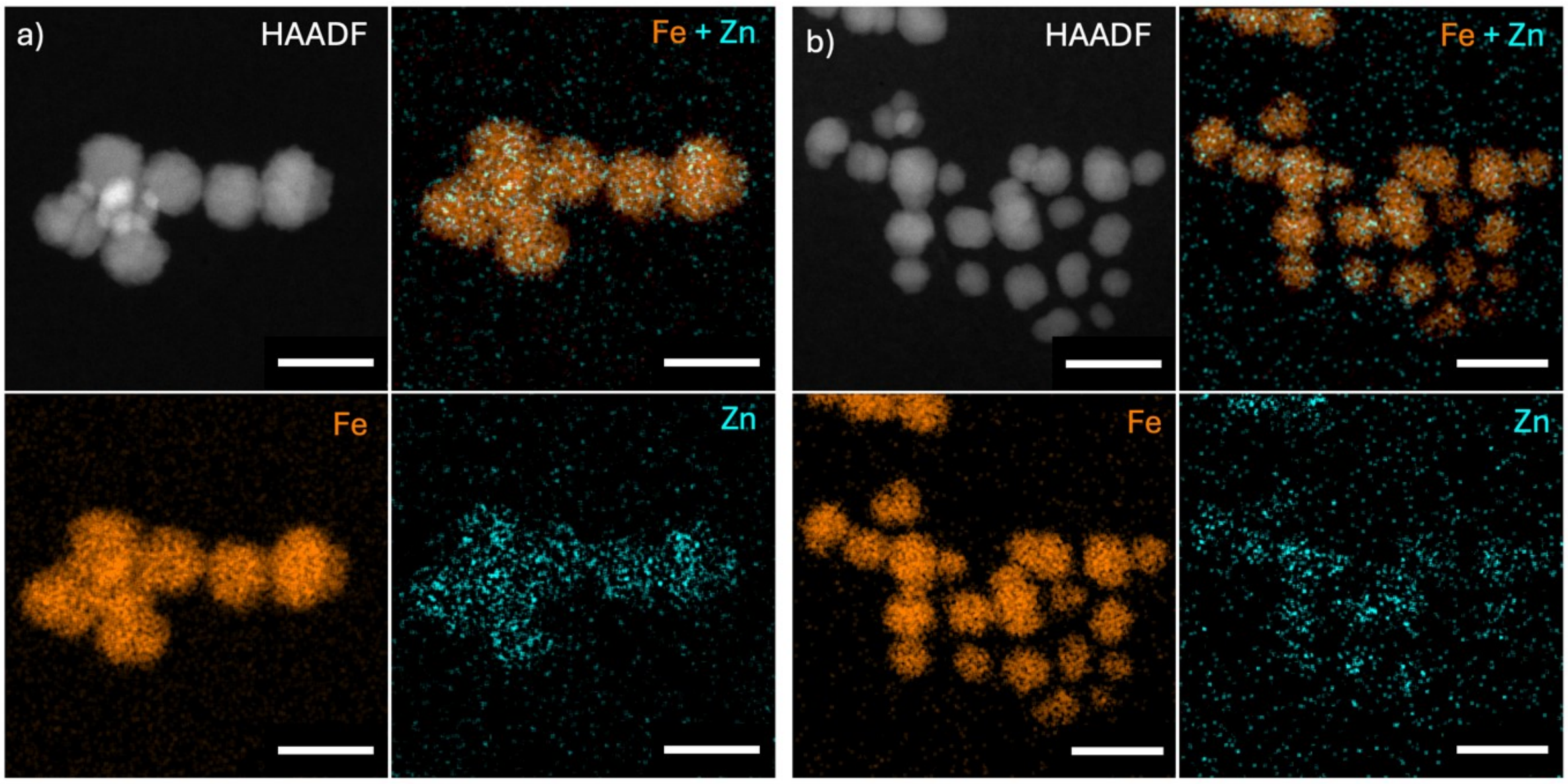

Figure S1. EDS elemental mapping of (a) 12 at. % Zn magnetite, (b) 12 at. % Zn maghemite, (c) 0 at. % Zn magnetite, and (d) 0 at. % Zn maghemite. The scale bar of each image is 20 nm.

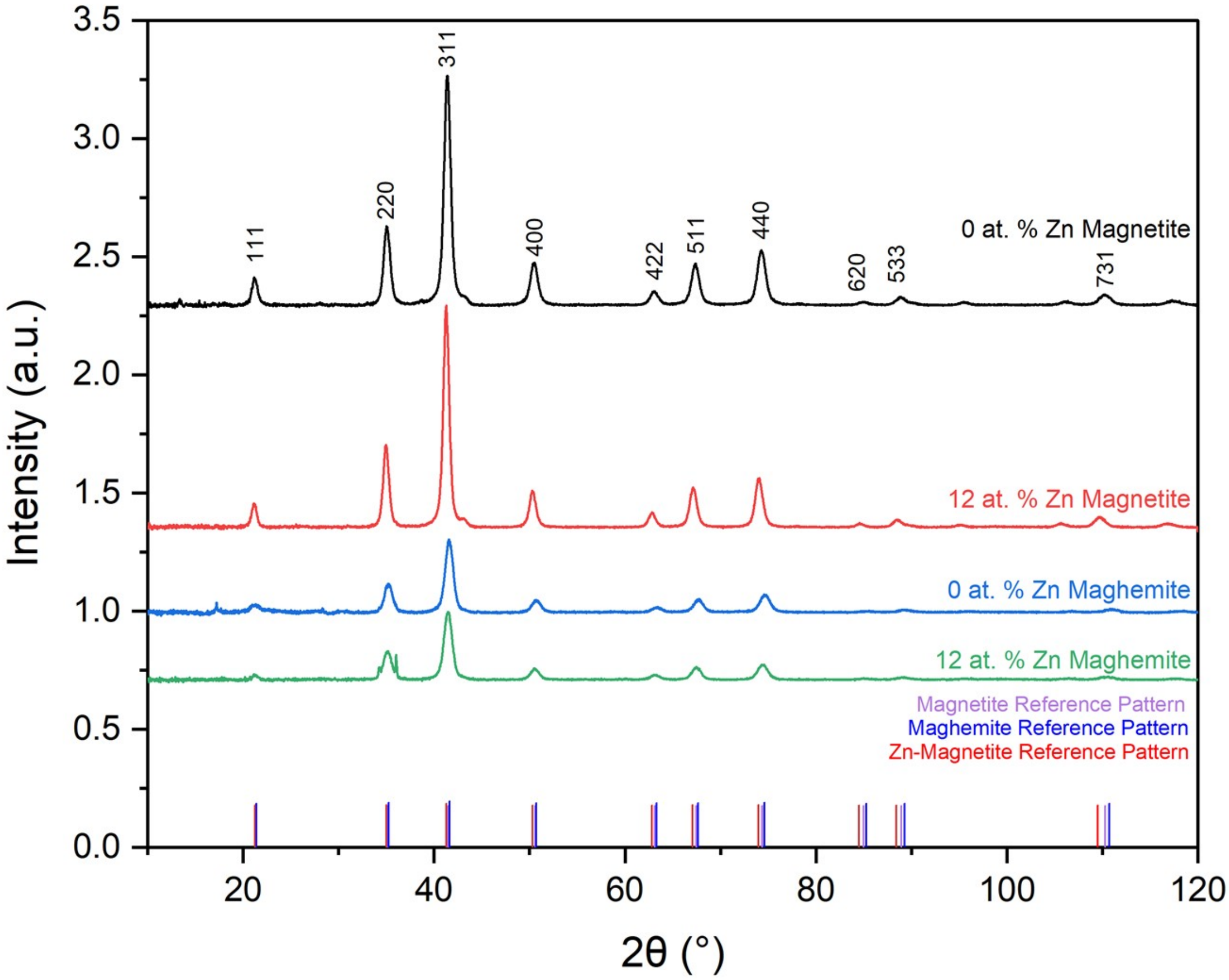

Figure S2. XRD patterns of synthesised magnetite and maghemite nanoparticles with varying compositions: 0 at. % Zn magnetite (black), 12 at. % Zn magnetite (red), 0 at. % Zn maghemite (blue) and 12 at. % Zn maghemite (green) compared with reference patterns for Magnetite (JCPDS Card No. 04-002-3668), $ZnFe_2O_4$ (JCPDS Card No. 04-008-5689) and Maghemite (JCPDS Card No. 00-039-1346).

Table S1. Composition of magnetite and maghemite nanoparticles. Average diameters and crystallite size of nanoparticles determined by TEM and XRD, respectively. The average lattice constant of both undoped and doped nanoparticles were determined from the (311) XRD peak.

**Nanoparticle Composition**	**Average diameter by TEM (d_{TEM}) (nm)**	**Average crystallite size (D_{XRD}) (nm)**	**Lattice Constant (Å)**
0 at. % Zn Magnetite	12.9 ± 1.8 nm	12.6 ± 1.7 nm	8.382
12 at. % Zn Magnetite	12.7 ± 1.5 nm	8.6 ± 1.7 nm	8.414
0 at. % Zn Maghemite	12.6 ± 1.5	13.2 ± 2.4	8.351
12 at. % Zn Maghemite	13.0 ± 1.7	10.4 ± 2.3	8.418

Table S2. Summary of all Magnetic Properties and MPI Relaxometry Data

**Nanoparticle Composition**	**M_{sat} (emu/g_{NP})**	**H_c (Oe)**	**M_r (emu/g_{NP})**	**FWHM (mT) [absolute]**	**Max PSF (absolute, normalised)**
0 at. % Zn Magnetite	76	4.3	1.7	26.2	0.73
3 at. % Zn Magnetite	52	4.2	1.8	38.2	0.54
7 at. % Zn Magnetite	67	4.2	1.3	16.0	0.85
12 at. % Zn Magnetite	71	4.2	2.7	21.7	0.95
0 at. % Zn Maghemite	70	3.6	1.0	18.4	0.61
3 at. % Zn Maghemite	68	4.3	0.6	32.3	0.45
7 at. % Zn Maghemite	54	3.8	0.7	20.1	0.71
12 at. % Zn Maghemite	72	3.5	0.8	22.4	1.0

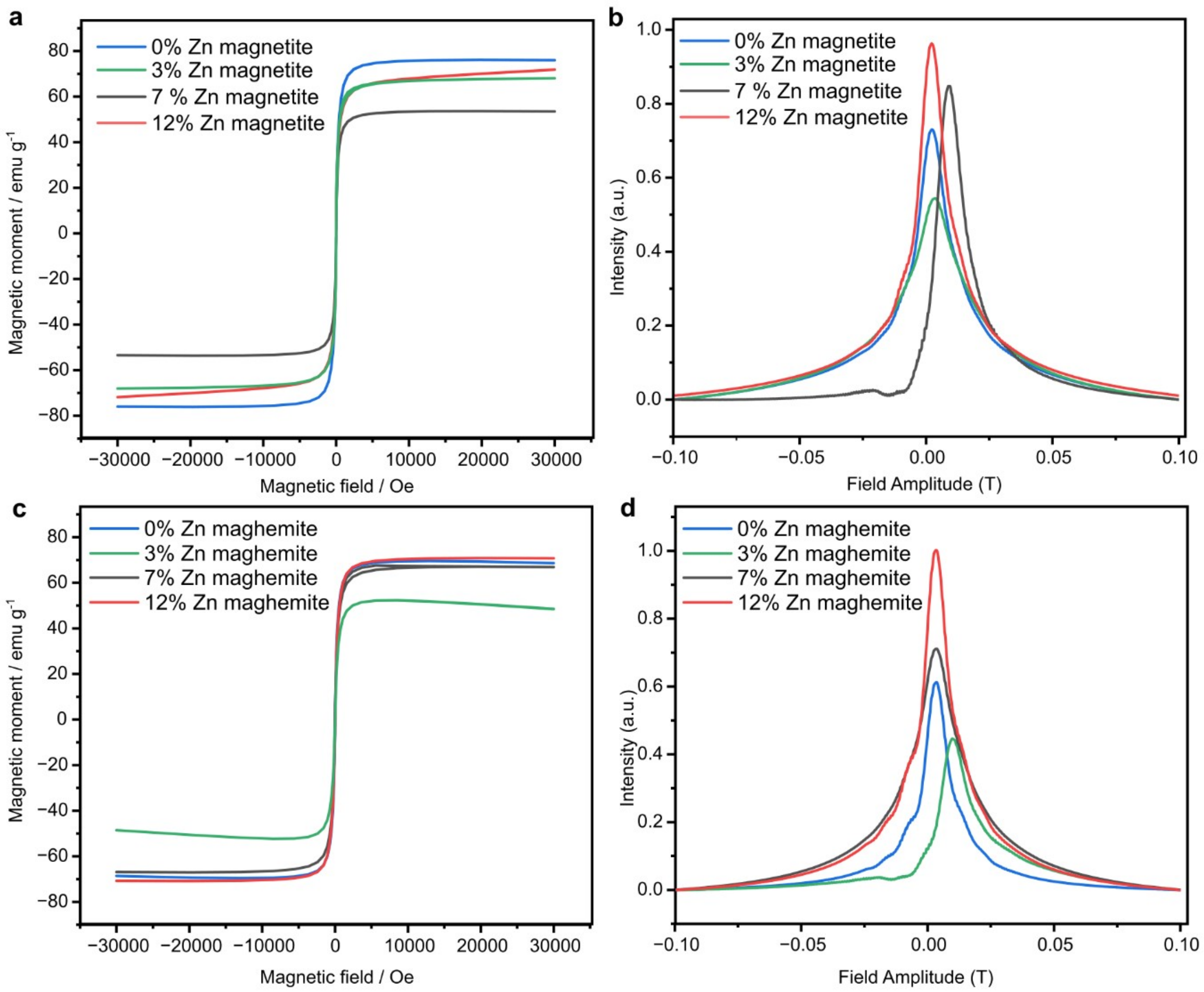

Figure S3. a,c) The hysteresis loop of magnetic nanoparticles at 300 K using vibrating sample magnetometer. b,d) Normalised point spread function. The value was normalised by the mass of the nanoparticle.

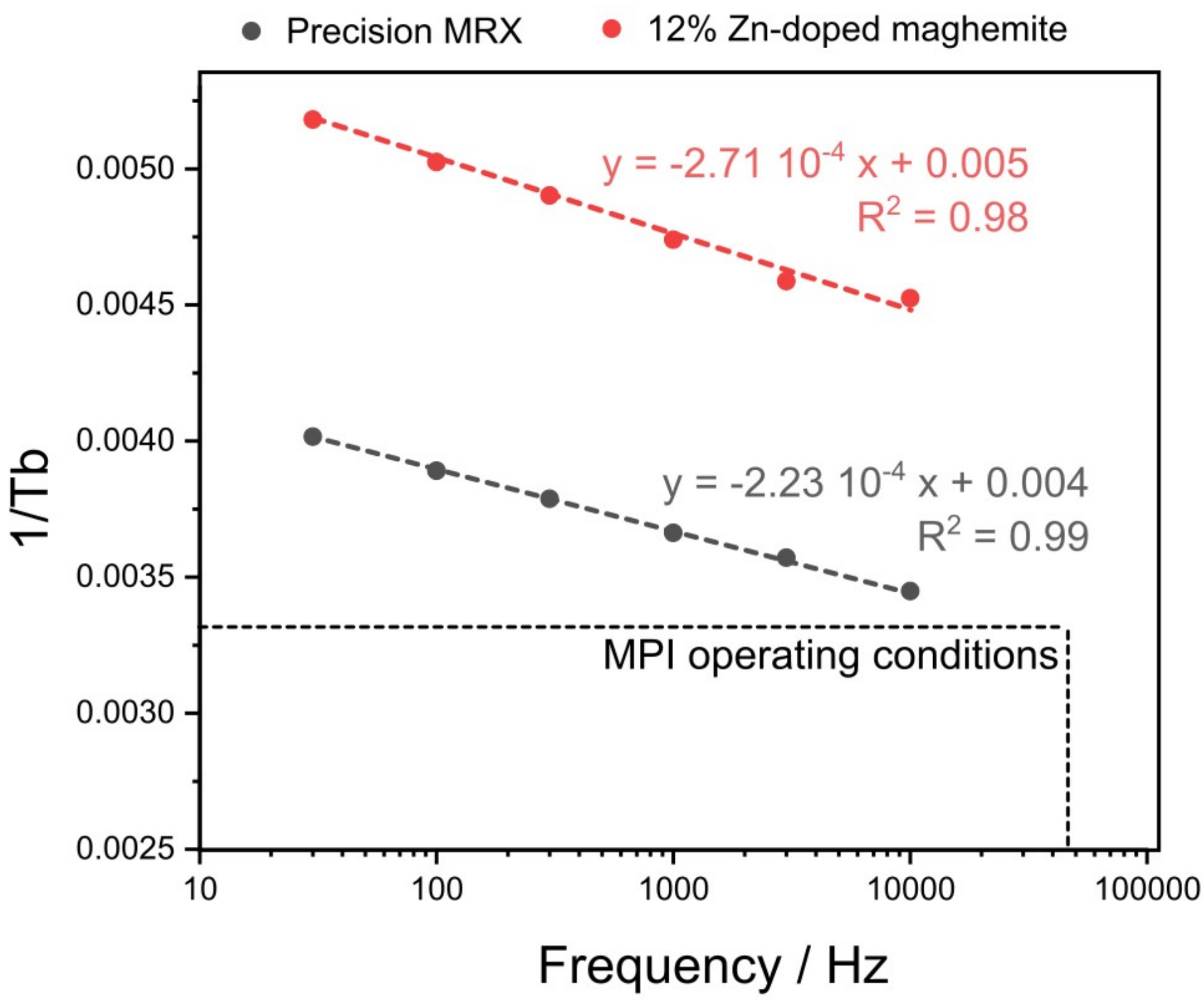

Figure S4. A plot of the reciprocal of blocking temperature against the frequency of 12 at.% Zn-doped maghemite and commercial PrecisionMRX® tracer. The linear lines indicate the frequency-dependent blocking temperature fits based on the Néel-Brown relaxation model.

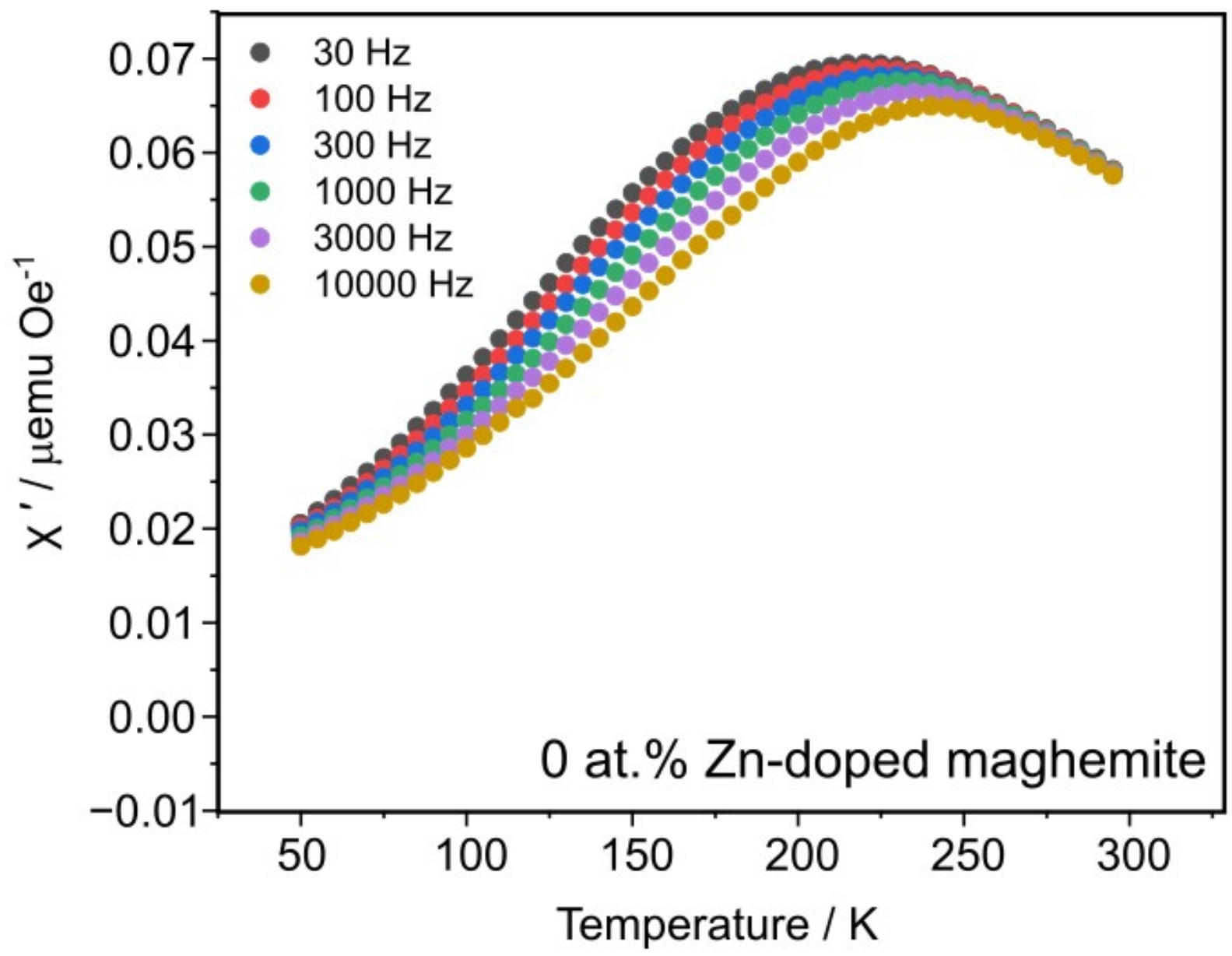

Figure S5. Alternating current (AC) susceptibility measurement of 0 at.% Zn-doped maghemite nanoparticles from 30 Hz to 10000 Hz.
